# Gene expression profiling in prognosis of distant recurrence in HR-positive and HER2-negative breast cancer patients

**DOI:** 10.18632/oncotarget.25258

**Published:** 2018-05-01

**Authors:** Tzu-Ting Huang, Nicolas Pennarun, Yu-Hao Cheng, Cheng-Fang Horng, Jason Lei, Skye Hung-Chun Cheng

**Affiliations:** ^1^ Department of Medical Research, Koo Foundation Sun Yat-Sen Cancer Center, Taipei, Taiwan; ^2^ Department of Medicine, Cathy General Hospital, Taipei, Taiwan; ^3^ Department of Product Development, Amwise Diagnostics Pte Ltd, Taipei, Taiwan; ^4^ Department of Radiation Oncology, Koo Foundation Sun Yat-Sen Cancer Center, Taipei, Taiwan

**Keywords:** breast cancer, distant recurrence prediction, microarray, gene expression profiling, chemotherapy

## Abstract

There had been several studies using gene-expression profiling in predicting distant recurrence in breast cancer. In this study, we developed an 18-gene classifier (18-GC) to predict distant recurrence of breast cancer and compared it with the 21-gene panel (Oncotype DX^®^, ODx) in performance. Included were 224 breast cancer patients with positive hormonal receptor (HR+) and negative human epidermal growth factor receptor 2 (HER2-). We compared the demographic, clinical, and survival information of the patients, and further compared the prediction of recurrence risk obtained by using the 18-GC with that by ODx. To have the best combined sensitivity and specificity, receiver operating characteristics (ROC) curve analysis was performed to determine the cutoff values for several breakpoint scores. For the new 18-GC, a breakpoint score of 21 was adopted to produce a combined highest sensitivity (95%) and specificity (39%) in detecting distant recurrence. At this breakpoint score, 164 of the 224 patients were classified by the 18-GC in the same risk level as by ODx, giving a concordance rate of 73%. Along with patient age and tumor stage, this 18-GC was found to be an independent significant prognostic factor of distant metastasis of breast cancer. We have thus created a new gene panel assay for prediction of distant recurrence in HR+ and HER2- breast cancer patients. With a high concordance rate with ODx, this new assay may serve as a good tool for individual breast cancer patients to make an informed decision on whether adjuvant chemotherapy should be performed post-surgery.

## INTRODUCTION

While breast cancer in Asia is characterized by a lower incidence rate than in the United States and Europe, [1, 2] it is still one of the leading causes of cancer death in Asia, particularly in Taiwan [3–5]. This disease is featured by its complexity due to the genetic heterogeneity of breast carcinomas [6]. Since a few decades ago, many gene-expression profiling studies of breast cancer have revealed the existence of four major subtypes differing markedly in prognosis: luminal-A, luminal-B, HER2-amplified, and basal-like [6], prevalence of which varies by racial/ethnic groups [7]. Among the Asian populations, prevalence of the luminal-A, luminal-B, HER2+/ER-, basal-like, and unclassified subtype has been shown to be 55-65%,, 10-20%, 10-15%, 10-15%, and 0-5%, respectively [8, 9]. In the current study, we focused on luminal-like breast cancer comprising the luminal-A and luminal-B subtypes, which are defined by the presence of hormonal receptor and absence of HER2 on the plasma membrane of tumor cells (i.e., HR+/HER2-) by the immunohistochemistry [10]. Several studies have suggested that luminal-like cancers tend to have the most favorable prognosis and longer-term survival when compared with the other subtypes [11, 12]. However, early-stage breast cancer patients with the luminal-like subtypes are commonly (up to 75%) overtreated with adjuvant chemotherapy despite that recent studies have indicated that adjuvant chemotherapy may not provide significant benefit in reducing risk of recurrence [13, 14].

To overcome this issue, several multigene panels, such as Oncotype DX^®^(ODx), MammaPrint^®^ and EndoPredict^®^ assay kits, have been developed to help clinical decision-making regarding adjuvant chemotherapy for patients with early-stage breast carcinomas. ODx (Genomic Health Inc., Redwood, CA) is a prognostic and predictive assay kit for women with HR+ and HER2- breast cancer. It is a 21-gene RT-PCR assay for 16 cancer-related and five housekeeping control genes with an aim to aid physicians and patients to determine the best course of treatment by predicting the risk of distant recurrence of breast cancer. The ODx assay produces a numerical recurrence score and places patients into three categories: low-, intermediate- and high-risk [15]. Another test (MammaPrint by Agendia BV, Amsterdam, Netherlands) is a microarray-based gene-expression profiling assay that can classify the risk of distant recurrence into two categories, low and high, by analyzing 70 genes of HR+ and node-negative patients that had not received adjuvant systemic therapy [16]. The third test, EndoPredict (Myriad Genetics, Salt Lake City, UT), is a 11-gene RT-PCR test that provides prognostic information regarding the risk of distant recurrence of breast cancer to patients with HR+ and HER2- tumors [17]. The assay measures the expression of eight cancer-related and three control genes, and classifies patients under endocrine therapy into low- or high-risk of distant recurrence.

Even though all such tools can help in making treatment decision on adjuvant chemotherapy for patients with early-stage breast carcinoma, none of them was originally developed for Asian patients even though Asian people may have different mechanisms in breast cancer due to multiple factors such as ethnic, environmental, and genetic variations [18–20]. To overcome such potential limitations, we developed an 18-gene classifier (18-GC) with tumor tissues obtained from Asian breast cancer patients and compared its performance with that of ODx in predicting distant metastasis in early-stage HR+/HER2- patients.

## RESULTS

### Patient characteristics

Two-hundred twenty-four HR+ and HER2- breast cancer patients were included in the study (Table 1) with 202 (90.2%) of the patients diagnosed with no metastasis during the development of their breast cancer and 185 (82.6%) treated with adjuvant chemotherapy. Among those receiving chemotherapy, 165 (89.2%) of the patients did not develop metastasis during their follow-up.

**Table 1:** Characteristics of patients with and without metastasis in HR+/HER2- invasive breast carcinomas (n=224)

Variable	Absence of metastasis	Presence of metastasis	p-value
	n=202	n=22	
	n (%)	n (%)	
**Age**			
≦40 (n=48)	38 (18.8)	10 (45.5)	0.011
>40 (n=176)	164 (81.2)	12 (54.5)	
**T stage**			
T1 (n=110)	106 (52.5)	4 (18.2)	0.003
T2-T3 (n=114)	96 (47.5)	18 (81.8)	
**N stage**			
N0 (n=130)	119 (58.9)	11 (50.0)	0.421
N1 (n=94)	83 (41.1)	11 (50.0)	
**Chemotherapy**			
No (n=39)	37 (18.3)	2 (9.1)	0.382
Yes (n=185)	165 (81.7)	20 (90.9)	
**Radiotherapy**			
No (n=93)	83 (41.1)	10 (45.5)	0.693
Yes (n=131)	119 (58.9)	12 (54.5)	
**Hormone therapy**			
No (n=9)	8 (4.0)	1 (4.5)	1.000
Yes (n=215)	194 (96.0)	21 (94.5)	
**Lymph vessel invasion**			
Nil/ Minimal (n=182)	166 (82.2)	16 (72.7)	0.263
Prominent (n=42)	36 (17.8)	6 (27.3)	
**ER status**			
Negative (n=5)	5 (2.5)	0 (0.0)	1.000
Positive (n=219)	197 (97.5)	22 (100.0)	
**PR status**			
Negative (n=40)	35 (17.3)	5 (22.7)	0.558
Positive (n=184)	167 (82.7)	17 (77.3)	

We found that two characteristics were significantly associated with the presence of metastasis in unadjusted analysis: a) an age at 40 or younger; b) a stage of T2-T3 (Table 1). Among the patients with no metastasis, 81.2% were over 40 years old, whereas only 54.5% of the patients with metastasis were categorized in the same age group (p=0.011). On the other hand, probability for patients with a T2-T3 tumor stage to develop metastasis is significantly higher than that for patients with a T1 tumor stage (81.8% with metastasis vs. 47.5% without metastasis for T2-T3 compared with 18.2% with metastasis vs. 52.5% without metastasis for T1, p=0.003).

### Determination of the breakpoint score

**Figure 1:**
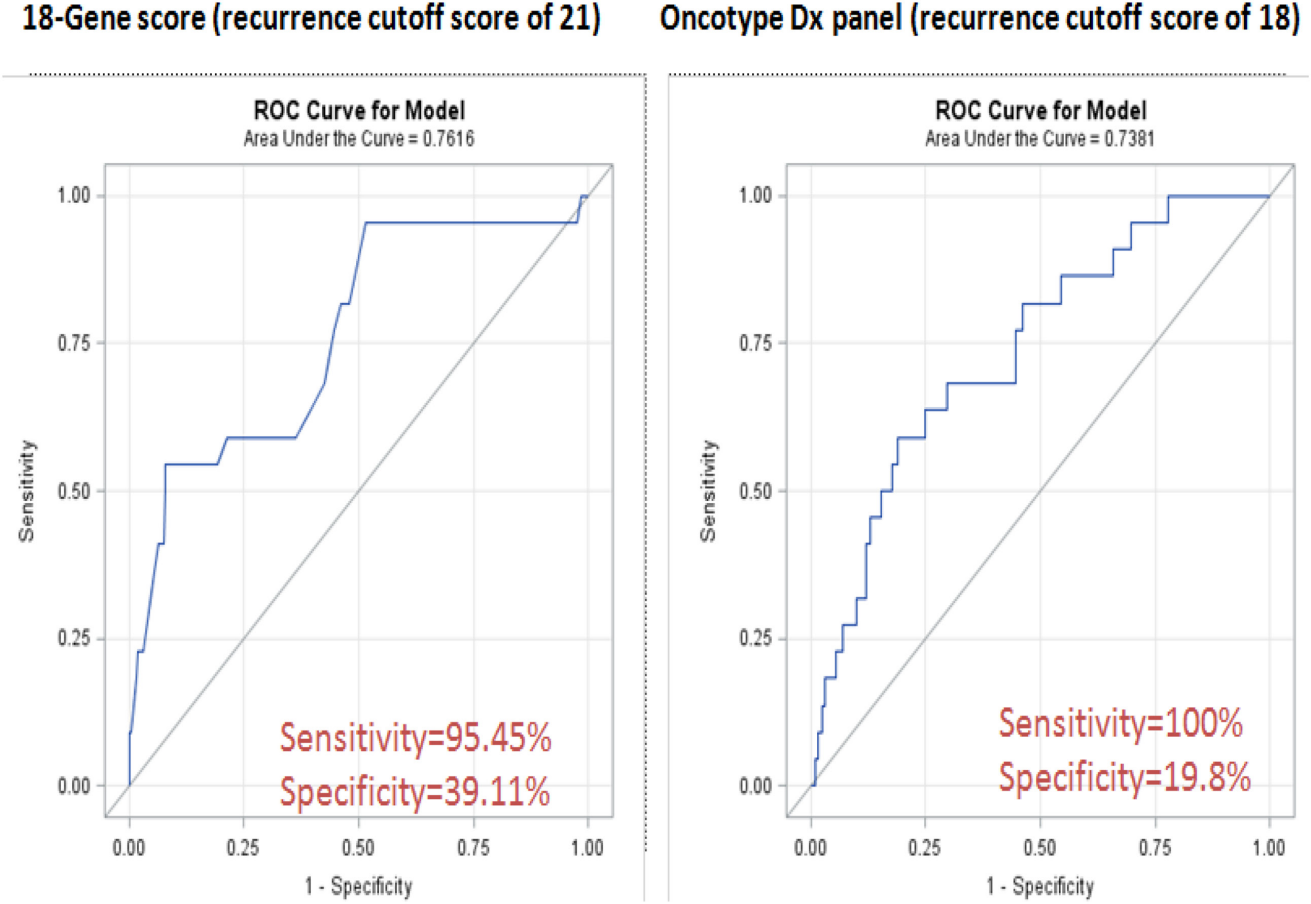
ROC curve analyses of the 18-GC and ODx

For the breakpoint determination in the original panel of the 18 genes selected previously (Table 2), we compared the ROC curves with different cutoff points that stratify patients into two groups: low- or high-risk of recurrence. We selected a breakpoint score of 21 (Figure 1) since this score minimized the distance on the ROC curve to the left top edge of the diagram and produced a combined greatest sensibility (95.4%) and specificity (39.1%). By using this breakpoint, 80 (35.7%) of the 224 patients were classified as having low risk and 144 (64.3%) high risk.

**Table 2:** Genes selected for gene-expression profiling analysis

Gene symbol	Gene title	GenBank accession number
TRPV6	Transient receptor potential cation channel, subfamily V, member 6	NM_018646
DDX39	DEAD (Asp-Glu-Ala-Asp) box polypeptide 39	NM_005804
BUB1B	Budding uninhibited by benzimidazoles1 homolog beta (yeast)	NM_001211
CCR1	Chemokine (C-C motif) receptor 1	NM_001295
STIL	SCL/TAL1 interrupting locus	NM_003035
BLM	Bloom syndrome	NM_000057
C16ORF7	Chromosome 16 open reading frame 7	NM_004913
PIM1	Pim-1 oncogene	NM_002648
TPX2	TPX2, microtubule associated	NM_012112
PTI1	Homo sapiens elongation factor 1-alpha 1	NM_001402
TCF3	Transcription factor 3 (E2A immunoglobulinenhancer binding factors E12/E47)	NM_003200
CCNB1	Cyclin B1	NM_031966
DTX2	Deltex 2, E3 Ubiquitin Ligase	NM_020892
ENSA	Endosulfine alpha	NM_004436
RCHY1	Ring Finger And CHY Zinc Finger Domain Containing 1, E3 Ubiquitin Protein Ligase	NM_015436
NFATC2IP	Nuclear Factor Of Activated T-Cells, Cytoplasmic, Calcineurin-Dependent 2 Interacting Protein	NM_032815
OBSL1	Obscurin-like 1	NM_015311
MMP15	Matrix Metallopeptidase 15 (Membrane-Inserted)	NM_002428

Patients classified in the intermediate-group by ODx were considered high risk when in comparison with the 18-gene panel. By using a breakpoint score of 21 in our gene panel assay, a total of 164 patients was classified in the same risk level as the ODx assay (73.2% concordance), indicating a significant agreement in the outcome predictions for individual patients (Table 3).

**Table 3:** Concordance between the 18-GC and ODx

	Risk group	18-gene classifier		
		Low (<21)	High (≧21)	Total
**Oncotype**	Low (<18)	30 (75.0%)	10 (25.0%)	40 (17.9%)
**DX**	High (≧18)	50 (27.2%)	134 (72.8%)	184 (82.1%)
	Total	80 (35.7%)	144 (64.3%)	224 (100%)

### Recurrence rate and recurrence-free survival by distant metastasis

To evaluate the prognostic power of the 18-GC, we compared the status predicted by the 18-GC and the actual distant metastasis status (Table 4). Even though the calculated PPV at 14.6 is relatively low, the calculated NPV is relatively high at 98.8%, indicating that the 18-GC is relatively accurate in identifying patients that would not have distant metastasis in the end of clinical monitoring.

**Table 4:** Concordance between predictions by the 18-GC and the actual clinical distant metastasis outcomes

	Distant metastasis			Total
	Risk group	No	Yes	
**18-gene classifier**	Low (<21)	79 (98.8%)	1 (1.2%)	80 (35.7%)
	High (>=21)	123 (85.4%)	21 (14.6%)	144 (64.3%)
	Total	202 (90.2%)	22 (9.8%)	224 (100%)

In addition, we calculated the PPV and NPV for patients who was not treated by chemotherapy (n=39). Results showed that the PPV is at 10% and the NPV at 100%, meaning that the 18 GC is well precise for determining the risk if a patient will have distant metastasis, especially for patients classified as low-risk (Table 5).

**Table 5:** Concordance between predictions by the 18-GC and the actual clinical distant metastasis outcomes for patients without adjuvant chemotherapy

	Distant metastasis			Total
	Risk group	No	Yes	
18-gene classifier	Low (<21)	19 (100%)	0 (0%)	19 (48.7%)
	High (>=21)	18 (90%)	2 (10%)	20 (51.3%)
	Total	37 (94.9%)	2 (5.1%)	39 (100%)

We then performed a univariate and multivariate Cox proportional-hazards analysis for the factors of age, tumor stage, lymph node status, tumor grade, lymph vessel invasion, and risk classification by the 18-GC (Table 6). In the unadjusted model, we found that age at diagnosis, tumor stage, and classification by the 18-GC are significant prognostic factors of recurrence-free survival by distant metastasis. In the adjusted model, age at diagnosis, tumor stage and the 18-GC remain significantly associated with recurrence. Patients at age 40 or younger upon diagnosis had worse breast cancer-recurrence survival (HR=3.2; 95% CI = 1.4 – 7.4) than those older than 40. Similarly, patients with T2-T3 breast cancer (HR=3.3; 95% CI = 1.1 - 10.2) had worse prognosis than those with T1 breast cancer. A score equal to or higher than 21 by the 18-GC is a significant factor of shorter recurrence-free survival (HR=11.7; 95% CI = 1.5 - 89.9) after adjustment for other clinical and pathological variables.

**Table 6:** Cox proportional hazard models for recurrence-free survival

Variable	Crude		Adjusted	
	HR (95% CI)	P-value	HR (95% CI)	P-value
**Age**				
≦40 (n=48)	3.2 (1.4 – 7.4)	0.007	2.7 (1.1 – 6.3)	0.025
>40 (n=176)	1 (Reference)		1 (Reference)	
**T stage**				
T1 (n=110)	1 (Reference)	0.009	1 (Reference)	0.039
T2-T3 (n=114)	4.3 (1.4 – 12.6)		3.3 (1.1 – 10.2)	
**N stage**				
N0 (n=130)	1 (Reference)	0.439	1 (Reference)	0.294
N1 (n=94)	1.4 (0.6 – 3.2)		0.6 (0.2 – 1.5)	
**Tumor grade**				
1 (n=59)	1 (Reference)	0.396	1 (Reference)	0.614
2 (n=106)	2.2 (0.6 – 8.0)		1.4 (0.4 – 5.0)	
3 (n=59)	2.4 (0.6 – 9.4)		0.8 (0.2 – 3.4)	
**Lymph vessel invasion**				
Nil/ Minimal (n=182)	1 (Reference)	0.232	1 (Reference)	0.298
Prominent (n=42)	1.8 (0.7 – 4.5)		1.7 (0.6 – 4.8)	
**18-gene classifier**				
<21 (n=80)	1 (Reference)	0.015	1 (Reference)	0.018
≧21 (n=144)	12.1 (1.6 – 89.9)		11.7 (1.5 – 89.9)	

Abbreviations: HR, hazard ratio; CI, confidence interval.

**Figure 2:**
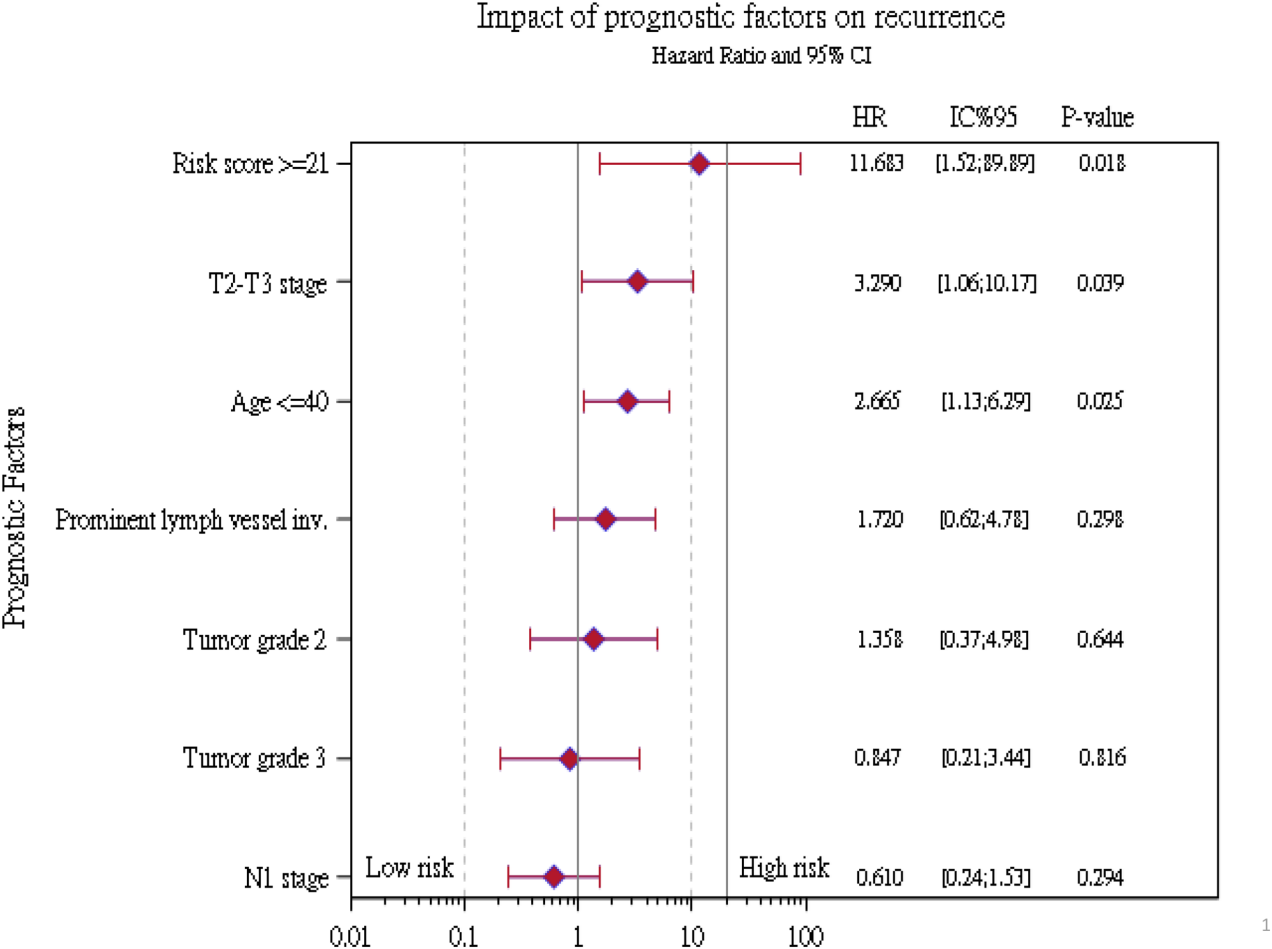
Summary of recurrence hazard ratios for different risk factors in subgroup analyses

A forest plot of the HRs obtained from exploratory subgroup analyses for recurrence-free survival is shown in Figure 2. The results indicate that prominent lymph vessel invasions, tumor grade, and lymph node stage were no longer significant prognostic factors in some subgroups. A possible reason for this observation is that N1 patient usually would receive more adjuvant chemotherapy and radiotherapy than N0 patients. In contrast, the 18-gene panel classification, T2-T3 stage, and age at 40 or younger remain significant factors and might be important confounders for recurrence prognosis in breast cancer.

**Figure 3:**
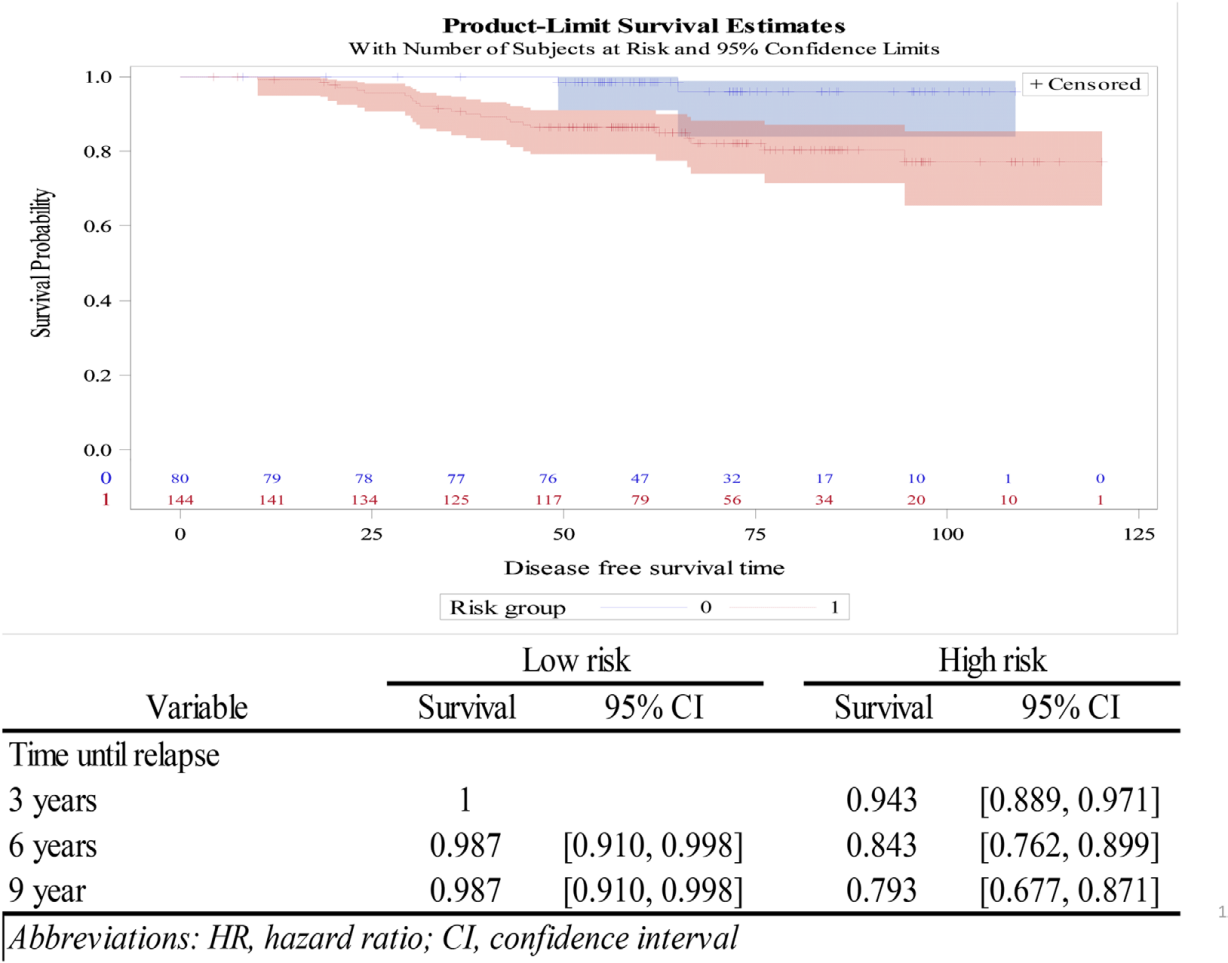
Survival plot analysis of low- and high-risk patients as determined by the 18-gene classifier

Recurrence-free survival rates were recorded at the 3-, 6-, and 9-year time points (Figure 3). It was found that patients classified as having low risk of recurrence by the 18-GC had high survival rates (0.960) even after nine years (Figure 3). In contrast, those classified as having high risk, the survival rates declined constantly over time and reached 0.772 at year nine (Figure 3). The survival probability of the low- and high-risk patients is plotted against the disease-free survival time.

## DISCUSSION

Early-stage patients with the luminal-like subtypes of breast cancer are commonly overtreated with adjuvant chemotherapy [14]. To overcome this issue, genomic assays, including ODx, have been utilized to predict the recurrence risk of early-stage breast cancer. They can help patients to avoid the potential adverse toxicity from chemotherapy when the recurrence risk and treatment benefit is low [24]. On the other hand, such prognostic genomic assays should not miss to identify patients that are at high risk of recurrence and could benefit from chemotherapy [25].

Nevertheless, there may be differences in gene expression profiles among different ethnic groups. For example, ODx has been shown to overestimate the risk of recurrence in Asian patients [26]. Even in the intermediate-risk patients, the 10-year distant metastatic rate is 0%. Furthermore, Japanese women have been reported to have better survival according to the SEER data between 1973-1994 [27]. A recent study has also shown that the breast cancer-specific deaths in stage-I patients at year 7 in white, black, and Asian women are significantly different (hazard ratios of 1, 1.57 and 0.60, respectively) [18]. It may thus be important to have a prognostic kit specifically for Asian breast cancer patients.

To that end, based upon an Asian breast cancer patient cohort, we have developed a kit for expression profiling of a panel of 18 genes with an aim to predict distant recurrence risk in luminal-like breast cancer patients. To test the prognostic power of this 18-GC, we included 224 patients and analyzed the association of its prediction with the actual metastasis outcome retrospectively. It was found that this 18-GC is a significant independent prognostic factor of distant metastasis (Table 5; Figure 2). Further, at the breakpoint score of 21, this 18-gene panel classified 164 patients into the same risk group as did the 21-gene ODx panel, giving a concordance rate of 73%. Despite the relatively high concordance rate between the 18-GC and the ODx assay, it is noteworthy that 50 out 80 patients classified as having low risk by the 18-GC were classified as having high risk by ODx. These patients would be recommended to receive adjuvant chemotherapy if based on the risk scores assigned by ODx. However, from the retrospective clinical outcomes, it was found that patients assigned as having low risk of distance recurrence by the 18-GC had a high (98.8%) distant metastasis-free rate (Table 4) and a high probability of a long recurrence-free survival (Table 6 and Figure 3), which contrasts with the phenomenon observed among luminal-like (both the luminal-A and -B subtypes) breast cancer patients whose survival decreases constantly over time even though they had been classified in the low-risk group by ODx [28]. Even though a prospective study with a larger patient cohort is warranted, the new panel that we developed may serve as a good tool for breast cancer patients, especially those with an Asian ethnicity, to make a personalized and informed decision on whether chemotherapy should be performed.

## MATERIALS AND METHODS

### Patient selection

Retrospectively we included in this study a total of 224 luminal-like (HR+/HER2- ) and T1-3N0-1 breast cancer patients treated at Koo Foundation Sun Yat-Sen Cancer Center (KFSYSCC) in Taipei, Taiwan between 2005 and 2012, for evaluation of the 18-GC developed in our institute [21]. The institutional review board of KFSYSCC reviewed and approved the protocol and informed consent documents for the study. Eligible patients had invasive breast cancer; surgery as first treatment (mastectomy or breast-conserving surgery); a positive test result for estrogen or progesterone receptors (HR+); a negative test result for HER2 (HER2-); a few positive lymph nodes between 0 and 3. Patients with an N2, N3 or M1 stage and treated with pre-operative chemotherapy were excluded.

### The 18-gene classifier

Development of the 18-GC has been reported previously [21]. Briefly, it was developed based on 135 breast cancer patients, including 112 patients treated in KFSYSCC, who developed no and 23 patients who developed LRR. Including a total of 18 recurrence-related genes, the 18-GC is a multifunctional gene panel that is associated with cell cycle and proliferation (DDX39, BUB1B, STIL, TPX2, CCNB1), oncogenic process (BLM, TCF3, PIM1, RCHY1, PTI1), inflammation and immune response (CCR1, NFATC2IP), cell-cell interaction (TRPV6, OBSL1, MMP15), apoptosis (C16ORF7, DTX2) and metabolism (ENSA) [21]. For risk classification, each gene was assigned a weight according to the Cox proportional hazards model to assemble the 18-gene scoring algorithm. With a range of risk scores between zero and 56, the breakpoint value of 21 was used to separate the low- from the high-risk category of distant recurrence. The algorithm of 18-GC is shown as below: [21]

18-gene score = 4 × TRPV6 + 3 × DDX39 + 8 × BUB1B + CCR1 + STIL + 3 × BLM + 11 × C16ORF7 + 4 × PIM1 + TPX2 + 2 × PTI1 + 2 × TCF3 + CCNB1 + DTX2 + 2 × ENSA + 5 × RCHY1 + 4 × NFATC2IP + OBSL1 + 2 × MMP15

Unlike ODx, we did not include an intermediate risk group because it is usually binary in clinic decision-making. By adopting the same statistical predictive model used by Paik et al [22], the raw recurrence score (*Xi*) is first calculated by using the following expression:

*Xi* = 0.47 × GRB7 group score - 0.34 × ER group score + 1.04 × proliferation group score + 0.10 × invasion group score + 0.05 × CD68 - 0.08 × GSTM1 - 0.07 × BAG.

The final recurrence score (*Yi*) was then calculated by transforming *Xi* using the following expression:

$$Yi=\left(Xi-5.1031\right)+100\times \frac{1}{10.7148-5.103}$$

### Prognostic factors

Along with the demographic and clinical variables previously identified with a prognostic value for distant recurrence (such as age at the diagnosis: ≦40 vs. >40 years old; tumor stage: T1 vs. T2-T3; lymph nodes: N0 vs. N1; tumor grade: grade 1 vs. grade 2 vs. grade 3; prominent vs. nil/focal lymph vessel invasion), the classification by our 18-GC (low vs. high risk) was included for analysis.

### Statistical analysis

The demographic, clinical, and survival information were collected and analyzed among patients with and without metastasis. Crude and adjusted Cox analyses were used to compare patients in the low- and high-risk groups assigned by the 18-GC. ROC curve analyses were then performed to identify the optimal breakpoint [23]. We then evaluated sensitivity, specificity, accuracy, negative predictive value (NPV), positive predictive value (PPV), and the area under the curve (AUC) to determine how well the new 18-GC prediction model performs as compared with the ODx assay. All the statistical analyses (p < 0.05) were performed using SAS Software, version 9.4.

## CONCLUSION

We have created an 18-GC for predicting the risk of distant recurrence in luminal-like breast cancer patients. Even though a study with a larger patient cohort conducted in a prospective way is warranted, the new 18-GC panel assay has the potential to become a good prognosis predictor for breast cancer patients, especially those of an Asian descent, to determine whether a given patient needs adjuvant chemotherapy.
